# Image Processing of Porous Silicon Microarray in Refractive Index Change Detection

**DOI:** 10.3390/s17061335

**Published:** 2017-06-08

**Authors:** Zhiqing Guo, Zhenhong Jia, Jie Yang, Nikola Kasabov, Chuanxi Li

**Affiliations:** 1 College of Information Science and Engineering, Xinjiang University, Urumqi 830046, China; gzqxju@163.com; 2 Institute of Image Processing and Pattern Recognition, Shanghai Jiao Tong University, Shanghai 200240, China; Jieyang@sjtu.edu.cn; 3 Knowledge Engineering and Discovery Research Institute, Auckland University of Technology, Auckland 1020, New Zealand; nkasabov@aut.ac.nz; 4 School of Physical Science and Technology, Xinjiang University, Urumqi 830046, China; lcxxju@163.com

**Keywords:** porous silicon microarray, feflected light image, pretreatment, tilt correction, spot segmentation

## Abstract

A new method for extracting the dots is proposed by the reflected light image of porous silicon (PSi) microarray utilization in this paper. The method consists of three parts: pretreatment, tilt correction and spot segmentation. First, based on the characteristics of different components in HSV (Hue, Saturation, Value) space, a special pretreatment is proposed for the reflected light image to obtain the contour edges of the array cells in the image. Second, through the geometric relationship of the target object between the initial external rectangle and the minimum bounding rectangle (MBR), a new tilt correction algorithm based on the MBR is proposed to adjust the image. Third, based on the specific requirements of the reflected light image segmentation, the array cells are segmented into dots as large as possible and the distance between the dots is equal in the corrected image. Experimental results show that the pretreatment part of this method can effectively avoid the influence of complex background and complete the binarization processing of the image. The tilt correction algorithm has a shorter computation time, which makes it highly suitable for tilt correction of reflected light images. The segmentation algorithm makes the dots in a regular arrangement, excludes the edges and the bright spots. This method could be utilized in the fast, accurate and automatic dots extraction of the PSi microarray reflected light image.

## 1. Introduction

At present, the process of commercial microarray (biochips) detection labels the target molecule or probe molecule with fluorescent markers. After this biological reaction, the fluorescent markers remain on the chip, and fluorescence can be generated by an excitation light [1,2,3,4]. This allows analysis of the sequence and quantities of target molecules by detection of the intensity distribution of the hybridization signal [5]. Up to now, commercial biological microarray detection has been done by fluorescence and isotope labeling techniques [6,7,8]. However, there are some problems in the detection methods of fluorescent labeling: conventional microarray and sequencing technologies often require several hours of manual operation to complete the labeling process, complex labeling is time-consuming, which leads to high costs, poor reproducibility, and difficulty of use in clinics. Due to the introduction of labeled molecules, it is possible to affect and change the structure and activity of biological molecules, often resulting in the test results having difficulty reflecting the interaction of biological molecules and the authenticity of the characteristics. Generally, the intensity of the excited fluorescence signal is very weak relative to the intensity of the excitation light, and the background noise is greatly disturbed [9]. Moreover, fluorescent microarrays have several problems in application; for example, the price of fluorescent markers is very high, and they are only semi-quantitative.

Based on the above reasons, label free detection technology has gradually become the important development direction in the field of biological analysis, a variety of biochip label free detection methods such as surface plasmon resonance (SPR), micro-ring resonators, ellipsometry, interferometry, scanning Kelvin probe (SKN), atomic force microscopy (AFM), nanowires and nanotubes, micro cantilever, and electrochemical impedance spectrum (EIS) have been proposed in recent years [10,11,12,13,14,15,16,17,18,19,20,21]. Recently, a high sensitivity and low costs label free biological detection method based on porous silicon microarray reflected light image is reported [22,23].

Porous silicon as an excellent biomaterial can be utilized to manufacture various kinds of photonic devices, which gives it excellent prospects for application to the field of biosensors [24,25,26,27,28]. The proposed PSi microarrays combine lithography technology with electrochemical etching. Each cell of the PSi microarray is a one-dimensional photonic crystal with defect layers (microcavities). If the biological reaction occurs in the microcavity, the refractive index will increase, which causes the defect state wavelength redshift [22]. For incident laser with a defect state wavelength, the reflectivity will increase. The change of reflectivity can be detected by the change of the image gray level of the PSi microarray. Therefore, based on the reflected light image of the PSi microarray, the biological response of each cell in the microarray can be quantitatively analyzed. The porous silicon microarray is different from the existing biochip based on measurement of fluorescence intensity of labels, and it can realize a high sensitivity refractive index detection only by detecting the gray value of the image of porous silicon microarray. Moreover, this detection is a parallel detection without a spectrophotometer. The image detection method based on the porous silicon microarray can be used to protein chips detection with low cost and high sensitivity.

The monocrystalline silicon array is produced by lithography technology. Each cell is a circular and equidistant arrangement. However, in the process of preparation PSi microcavities, electrochemical etching causes the edge of each circular cell to become irregular. After laser irradiation, the image produces complex noise. These unique characteristics are different from fluorescent images, which makes image processing difficult. In addition, the optical structure of the cell edge region is different from the central region, which results in different reflectivity rates from the cell edge region to the central region [23]. Therefore, it is necessary to remove the edge of each cell (including bright spots) by segmentation. Based on the aforementioned problems, the reflected light image is preprocessed in this paper, and the reflected light image is corrected and segmented on the basis of pretreatment, whereas a new method for dots extraction based on the reflected light image of PSi microarray is developed.

## 2. Acquisition of Porous Silicon Array Reflected Light Image

**Figure 1:**
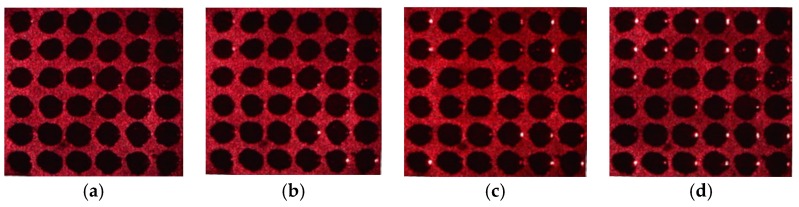
Microarray images obtained by digital microscope as a laser produces incident at different angles. (**a**) 0°; (**b**) 3°; (**c**) 5°; (**d**) 7°.

PSi chip arrays are produced on monocrystalline silicon by lithography technology and electrochemical etching. The diameter of each circular cell on the chip array is approximately 500 μm, and every circular cell has a microcavity structure [23]. By careful design of the parameters of the microcavity structure, when a He-Ne laser with a wavelength of 633 nm produces vertical incidence (0°), the minimum reflectivity is obtained. The laser incident angle starts from 0°, as the incident angle increases, the reflectivity will increase, as shown in Figure 1. This process is equivalent to the increase of the refractive index of the PSi microcavity when the laser produces vertical incidence [29]. Calculation and experimentation have proved that the average gray value of the array cells is directly proportional to the change of the refractive index [22]. Therefore, the change of the refractive index can be obtained by the change of the gray value of the cell in the image. This method has high detection sensitivity and can detect refractive index changes of less than 10^−3^ [22].

## 3. Extracting the Dots in Reflected Light Image

Before the fluorescent image analysis, it is usually necessary to perform tilt correction and spot segmentation [30]. At present, there are some tilt correction algorithms and segmentation algorithms based on fluorescent images, but these algorithms are not very suitable for reflected light images [31,32,33,34]. Because of the irregular shapes of the array cells and complex background in the reflected light image, it is necessary to develop processing algorithms that are suitable for the reflected light image. The processing algorithms should include three parts: pretreatment, tilt correction and spot segmentation.

### 3.1. Pretreatment

Many binarization algorithms have been proposed for image processing. Examples include the minimum error threshold method, the maximum entropy threshold method, the iterative threshold method and the maximum between class variance method. The minimum error threshold method is based on Bayes theory [35,36]. As a result, the binarization problem is transformed into a minimum error Gauss distribution fitting problem. The maximum entropy threshold method is to find the optimal threshold for binarization by measuring the entropy of the image histogram [37]. However, this algorithm has a slow computing speed and only considers the gray information of pixels. Therefore, the segmentation effect is unsatisfactory when the signal-noise ratio (SNR) is low. The iterative threshold method is based on the idea of approximation [38]. The initial threshold is continuously updated in the light of a strategy until a given constraint is satisfied. However, the threshold calculated with this method is not accurate enough. The maximum between class variance method divides the image into two categories according to the threshold value, and the optimal threshold is obtained when the variance between classes is the largest [39]. This algorithm was created to compensate for the deficiencies of the iterative threshold method, and it results in automatic, accurate, and fast threshold selection.

The background of fluorescent image is single, and the contrast between the spots and the background is very large. Therefore, the binary image can be processed directly by most binarization algorithms. However, the formation of speckle noise and impulse noise produced by the interaction between the laser and the rough surface of PSi bring great difficulties to binary image processing. To eliminate the interference of background and light intensity and lay a foundation for subsequent tilt correction and spot segmentation, binarization is a very important step. Thus, this paper presents a special pretreatment method.

The RGB (Red, Green, Blue) model is an inhomogeneous color space. The three components of the model have a high correlation. The value of each pixel is represented by the brightness value of red, green, and blue, and the brightness value will change with different illumination intensities. Therefore, the RGB color space is susceptible to changes in illumination conditions. It is very difficult to carry out binarization of the reflected light image.

The HSV model is a color space that is relatively close to the color perception of human eyes. The color characteristics of each pixel are represented by three components: hue, saturation and value, in which hue and saturation contain the color information of the image and value represents the luminance information affected by illumination conditions [40]. For the reflected light image, HSV space can remove the correlation among components. It is necessary to combine the saturation component and the value component in binarization processing.

**Figure 2:**
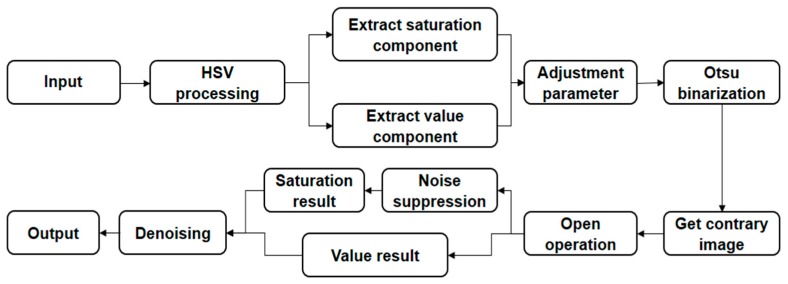
Algorithm flowchart.

The pretreatment method proposed in this paper first converts the image from RGB color space to HSV color space. The saturation component and value component are then extracted and normalized. The contrast and color of the image are changed by adjusting the parameters of the component. The maximum between class variance method is selected to accomplish the binarization of the saturation component and the value component—obtaining the opposite image in the meantime. By filling the holes in the image with morphological operations, the binarization results of the saturation component and the value component are obtained. In addition, the saturation component suppresses the bright object connecting to the image boundary, and the value component is not required. Because the saturation component represents the color distribution, the white area range of each cell is smaller than the actual range in the process, making it difficult to connect with the boundary. Therefore, boundary noise can be eliminated by suppression. The value component represents the gray level distribution, which can reflect the actual size of the cell. However, due to a complex background, it is easy to make the cells connect to the boundary after binarization. If the suppression means are taken, cells will be lost. Thus, it is better to use the results of the two components to judge the range of the cells and remove irrelevant noise in the background, which can achieve a better boundary segmentation effect. The processing area is traversed to determine the reasonableness of the region. Then, segmentation is performed for large areas, and small white noise and white parts connected to the boundary are removed. Finally, the binarization processing of the reflected light image is completed, which lays the foundation for realizing the following algorithms. The algorithm flowchart is shown in Figure 2.

### 3.2. Tilt Correction

Tilting of the image can be caused by several factors, such as inaccuracy of the position of the spots caused by the machine, error in slide installation angle in the microarray preparation, and scanner errors. This tilt can cause trouble in subsequent image processing. Therefore, it is very important for microarray images to undergo tilt correction.

In the field of fluorescent image correction, many tilt correction algorithms have been proposed, such as the Radon transform, Hough transform, Fourier transform, and Projection methods [41,42,43]. These algorithms are constantly being improved [44,45,46]. However, although these algorithms achieve automatic image calibration, there are still many problems. For example, in the Radon transform method, the tilt angle is obtained by projecting the images at different angles [44]. This algorithm not only has a large amount of computation, but also experiences calibration failure for some images due to energy distribution and prominent diagonals. Furthermore, the Power Spectrum Estimation Method must perform Fourier transformation at each step, and a huge amount of computing limits correction speed [45]. Some scholars have previously proposed that the correction angle can be calculated by the variance of projection waveform [46]. However, in the process of optimization, a large number of calculation is required in projection and variance. To achieve the same accuracy, the computational efficiency is very low.

In this paper, a new tilt correction algorithm based on MBR is proposed. The MBR is a simple algorithm for representing the size and shape of an object in the direction of the principal axis. At present, there are some common methods for extracting MBR, such as the rotating target method and the vertex chain code method [47,48]. In general, the result of the rotating target method depends on the size of each rotation angle. As the rotation angle decreases, the number of rotations increases, resulting in a longer computation time. Therefore, it is not easy to obtain an accurate MBR. The vertex chain code method has a faster computation time than the rotating target method, but the amount of calculations is still very large. 

**Figure 3:**
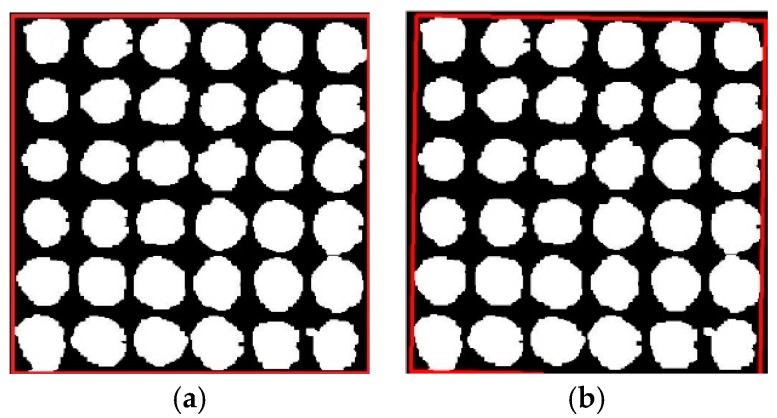
MBR extraction process. (**a**) initial external rectangle; (**b**) minimum bounding rectangle.

In this paper, the method of seeking the principal axis is used to obtain the MBR [49]. The binarization microarray image is scanned from the horizontal and vertical directions to determine the initial external rectangle, as shown in Figure 3a. The centroid of the rectangle is used as the center of rotation, and the horizontal and vertical axes are made to intersect each other at the center to determine the location of the initial principal axis. Through constantly rotating and translating the principal axis, the interval of the rotation is defined in an acute angle area that consists of two axes. The principal axis must be moved after every rotation. The maximum coordinates ($x_{max}$, $y_{max}$) and minimum coordinates ($x_{min}$, $y_{min}$) on rectangle boundary of each coordinate direction are recorded. From this, the corresponding rectangular area can be acquired with S. The formula is as follows:

(1) $$S=\left(x_{max}-x_{min}\right)\times \left(y_{max}-y_{min}\right).$$

When rotated to a certain angle, the area of MBR can be obtained. The rotation angle is the angle of the image tilt. The MBR is shown in Figure 3b.

**Figure 4:**
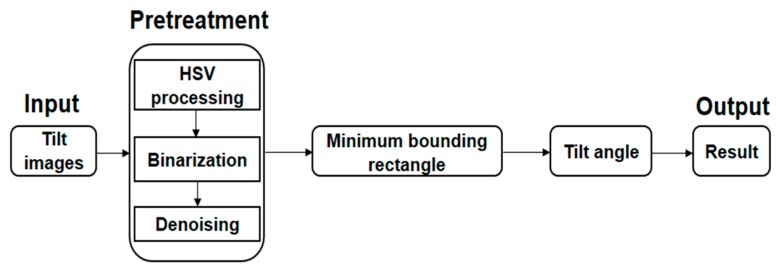
Algorithm flowchart.

The MBR correction algorithm consists of three parts. In the first part, the binarization image is acquired by preprocessing the tilted image. In the second part, the method of seeking the principal axis is used to get the MBR of the binarization image. In the third part, the tilt angle of the microarray image is calculated from the initial external rectangle to the MBR. The correction algorithm flowchart is shown in Figure 4.

### 3.3. Spot Segmentation

In order to obtain the position of the target spots, spot segmentation is a key step in microarray image processing. It has great significance for subsequent image analysis. In fluorescent images, spot segmentation is generally used to divide the real size of the spots [50,51]. At present, there are many segmentation algorithms based on that principle. These can be divided into different types, particularly fixed circle segmentation, adaptive circle segmentation, threshold segmentation and segmentation algorithms based on specific theories.

The fixed circle method uses a circular template that is similar to the spot size to achieve spot segmentation. This method was first used in Scan Alyze, a gene chip software developed by Stanford University, Stanford, CA, USA. It can segment simply and quickly by setting only the corresponding parameters. However, the size, shape and location of the spot have certain limitations, and the applicability is poor. Adaptive circle segmentation is improved on the basis of fixed circle segmentation. According to the size and location of different spots, a circular template with different radius and adjustable positions can be selected. Gene Pix Pro software (v6.0, Molecular Devices, Sunnyvale, CA, USA) used this method to accomplish spot segmentation. This method greatly improves the fixed circle method in terms of accuracy and applicability. However, in actual application, not all points are circular or approximately circular, and using only a circular template often results in the background area being divided into the target area. Threshold segmentation is one of the most common methods for fluorescent image segmentation. The key to threshold segmentation is to select different thresholds to achieve the ideal segmentation effect. This method is fast and not limited by the shape of the spots. However, when dealing with fluorescent images with low contrast, complex background and non-uniform fluorescence intensity of each point, the segmentation accuracy is poor, and spots are easily lost. Segmentation algorithms based on specific theories mainly include segmentation algorithms based on the Seeded Region Growing Method and the Morphological Watershed Algorithm [52,53]. Compared with other methods, these algorithms have high complexity, but the segmentation is accurate, and they are more suitable for the segmentation of fluorescent images.

However, none of the above image segmentation algorithms can be applied to reflected light images. Because of the different photonic structures between the edge region and the central region of the reflected light image, the edge needs to be cut during spot segmentation. The background of the reflected light image is complicated, which brings great difficulty to segmentation. In order to reduce errors in calculating the average gray value, it is necessary to take as many pixels as possible in calculation process. That is to say, it is in the scope of the cell to segment dots as large as possible and exclude the bright spot on the right side and edge part of the cell.

**Figure 5:**
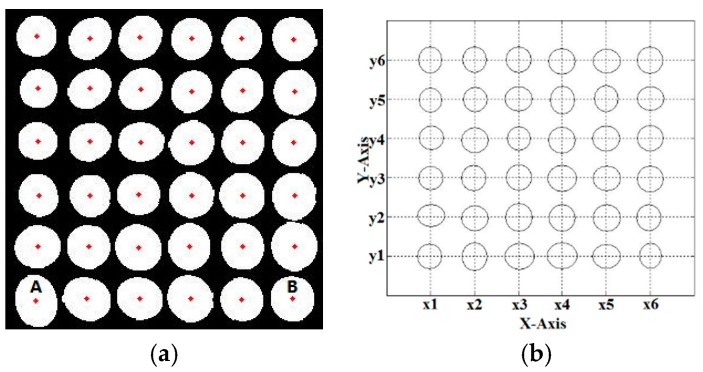
(**a**) extracting centroid coordinates; (**b**) determining the location of center coordinates.

In order to address the above problems, a new segmentation algorithm is proposed in this paper. The reflected light image after rotation is binarized according to the method of the pretreatment stage. At this time, the edge contour is irregular, making it difficult to accurately reflect the scope of cells. It is necessary to take the ellipse fitting on cells based on the least squares method [54]. Then, the smooth boundary contour of the cells can be acquired. The image is segmented on the basis of extracting the edge contour of the cells. As shown in Figure 5a, at first, the ellipse fitting of the binarization image is performed after pretreatment. Then, regional analysis is carried out, and the centroid coordinates of each cell are recorded. In order to subsequent image analysis, it is necessary to make the interval and radius of dots equal after segmentation. Firstly, the center position must be determined. It is known that A and B are two points in the first line of Figure 5a. The abscissa is discretized and divided equally so that the horizontal distance between points is equal. Then, it can determine the abscissa of each column with *X*_1_, *X*_2_, *X*_3_, *X*_4_, *X*_5_, and *X*_6_. Secondly, all of the ordinates of the first row are averaged to acquire ordinate Y_1_. The points are placed in a row on the same lines and calculated according to formula (2). Then, the ordinate of each line is determined with *Y*_1_, *Y*_2_, *Y*_3_, *Y*_4_, *Y*_5_, and *Y*_6_. The result is shown in Figure 5b, and the distance of all center coordinates is equal:

(2) $$Y_{n}=Y_{1}+\left(X_{n}-X_{1}\right).$$

**Figure 6:**
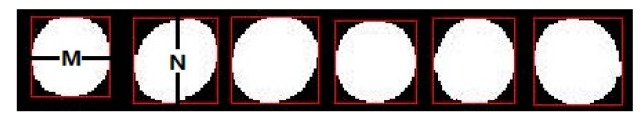
The horizontal and vertical distances.

Then, the radius of segmentation is determined according to the rectangular box of the white cell area to determine the horizontal distance signed M and the vertical distance signed N, as shown in Figure 6. In order to ensure that the segmentation results are in the range of all cells, the algorithm divides the minimum value of the horizontal and vertical distance of each cell by 2 to obtain the circular radius. In order to eliminate the bright spots on the right side of the cells, the algorithm uses the method of multiplying by 0.9 to acquire the reduced radius for segmentation.

**Figure 7:**
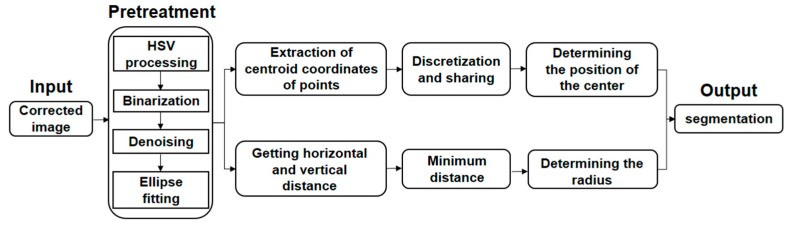
Algorithm flowchart.

The segmentation algorithm flowchart is shown in Figure 7.

## 4. Experimental Results and Analysis

In order to test the performance of the above algorithms, an experiment is carried out on the MATLAB platform (2014a, MathWorks, Natick, MA, USA). The research uses eighteen pictures that are divided into two categories: (1) the reflect angle varies from 0° to 8°, the number of rows and columns is 6 × 6, and the average size is 283 × 287 pixels, and (2) the reflect angle varies from 0° to 8°, the number of rows and columns is 12 × 12, and the average size is 567 × 576.

### 4.1. Pretreatment

**Figure 8:**
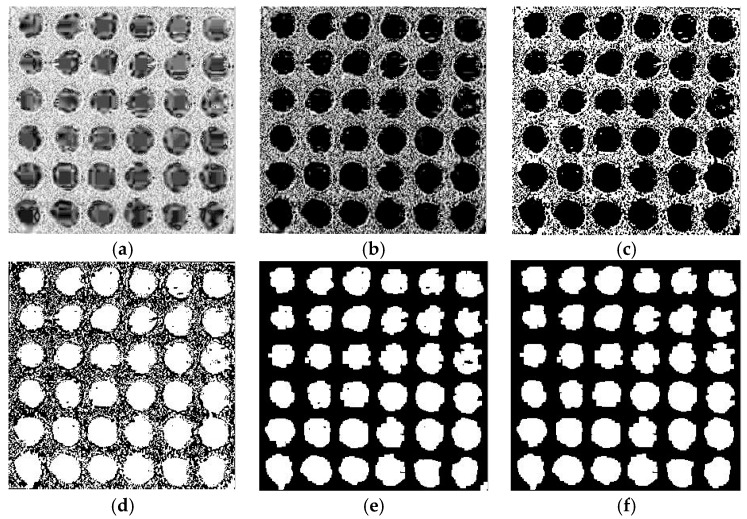
(**a**) saturation component; (**b**) adjusting saturation component; (**c**) binarization of saturation component; (**d**) acquiring the opposite image; (**e**) open operation; and (**f**) results of saturation component processing.

First, it is necessary to extract the saturation component, and the extraction result is shown in Figure 8a. In order to change the contrast and color of the image, it is necessary to adjust the parameters of the saturation component, and this adjustment result is shown in Figure 8b. The maximum between class variance method was selected to make achieve binarization of the saturation component on the adjusted image, and the result is shown in Figure 8c. After acquiring the opposite image and taking open operation, the results are shown in Figure 8d,e. At the same time, there were still some holes in the binary images that needed to be filled by morphology, and the bright objects connecting to the image boundary were suppressed. The processing result of the saturation component is demonstrated in Figure 8f.

**Figure 9:**
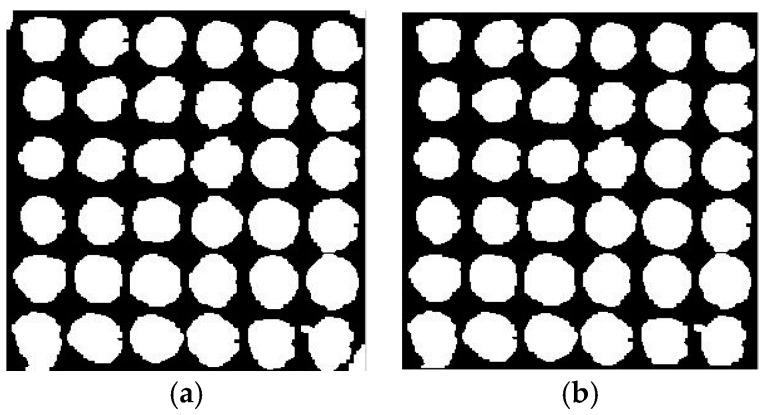
(**a**) processing result of value component; (**b**) removal of boundary noise.

The value component was extracted from the HSV space of the original image. The same steps given above were applied to the value component. However, it is not necessary to suppress the bright object connecting to the image boundary, and the result is as shown in Figure 9a. The edge of Figure 9a still has some irrelevant white areas, so it is necessary to remove the irrelevant white areas by combining the processing results of the saturation component. Segmentation was taken for large areas, and small white noise and white parts connecting to the boundary were removed. The pretreatment result is shown in Figure 9b.

The experimental results show that the pretreatment method proposed in this paper can successfully eliminate background and light intensity interference. The cells are divided to lay the foundation for the subsequent tilt correction and spot segmentation.

### 4.2. Tilt Correction

**Figure 10:**
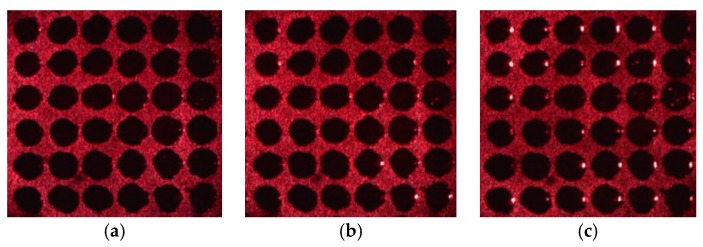
(**a**) 0°; (**b**) 3°; (**c**) 7°.

The experiment selected three reflected light images for which the reflection angle was 0°, 3°, and 7°. The actual tilt angles of the images were 0.98°, 0.90°, and 1.20°. The MBR correction algorithm automatically detected the angles as 0.92°, 0.63°, and 1.27°. The error was 0.06°, 0.27°, and 0.07°. The effect of the algorithm for image tilt correction is shown in Figure 10a–c.

Compared with the fluorescent image, the background is more complex and the interference is more significant in the reflected light image. The general fluorescent image correction algorithm is not suitable for the reflected light image. In order to reflect the performance of the proposed algorithm, the Radon transform correction algorithm was applied to the reflected light image for the contrast test [44]. The range of the algorithm used in the experiment was [−30°, 30°], with 0.01° as the step of the rotation projection, without using the small range. This is because a small projection range will result in robustness reduction and calibration failure. Additionally, by using the range of [−30°, 30°], the result of projection is a uniform distribution, which can reflect the actual tilt angle, enhance the robustness and adapt to a large number of images.

The data statistics of the two algorithms tested on the MATLAB platform are as shown in Table 1 and Table 2. The experimental pictures were reflected light images with nine pictures measuring 6 × 6 and nine pictures measuring 12 × 12. The test data of the average error and the average time was as shown below.

**Figure 11:**
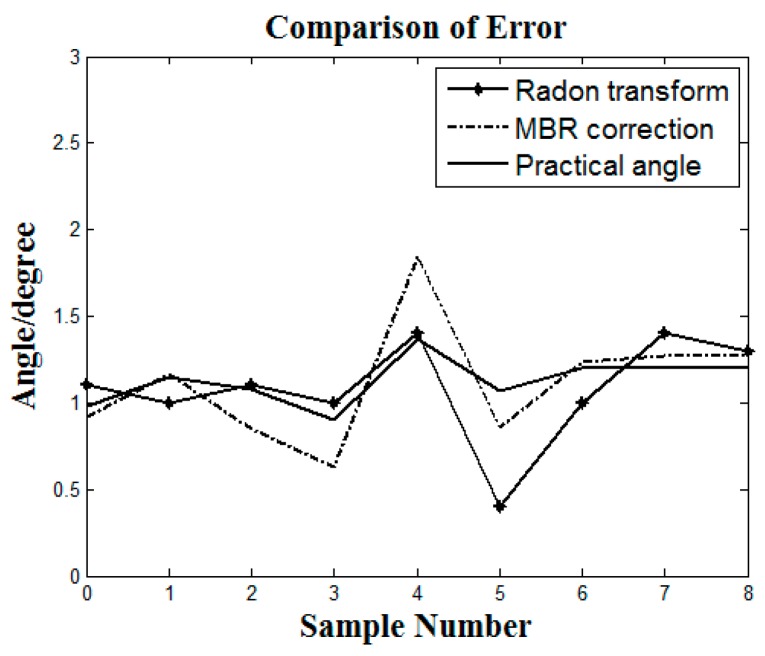
Comparison of error.

Figure 11 shows the error comparison of the two algorithms in dealing with the same experimental samples. The *x*-axis corresponds to nine test images and the *y*-axis is the angle of algorithm automatic detection. According to the experimental results, it is clear that the MBR correction algorithm shows good applicability and has an advantage in computing time for reflected light images processing. Compared with Radon transform, the algorithm’s error fluctuation is smaller, making it more suitable for the tilt correction of reflected light image.

The results indicate that, for the reflected light images, the proposed correction algorithm has the advantages of high calculation speed and low error. Especially when the reflected light image is large in size, the computation time of other algorithms can multiply; however, the computation time only increased slightly when the proposed algorithm was presented with large reflected light images. This algorithm can achieve fast and accurate tilt correction.

The experimental results show that the proposed correction algorithm also has a good effect on the fluorescent image.

### 4.3. Spot Segmentation

**Figure 12:**
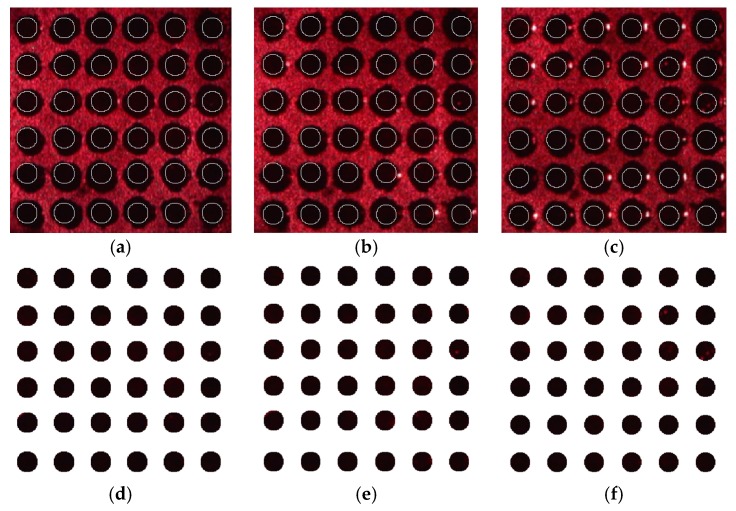
Segmentation results. (**a**) 0°; (**b**) 3°; (**c**) 7°; (**d**) 0°; (**e**) 3°; (**f**) 7°.

After binarization with the corrected images by the preprocessing method, and the ellipse fitting is performed. The center and radius of the circle can be obtained on the basis of binary image. Then, segmentation is accomplished. The experiment continuously selects three reflected light images with reflection angles of 0°, 3°, and 7°. The segmentation results are shown in Figure 12a–c. For the above images, the results of removing the background and extracting the segmented dots are separately shown in Figure 12d–f.

The average time of segmentation from the RGB image corrected to completion of segmentation is shown in Table 3. The experimental pictures were reflected light images; nine pictures were 6 × 6 and nine pictures were 12 × 12.

**Figure 13:**
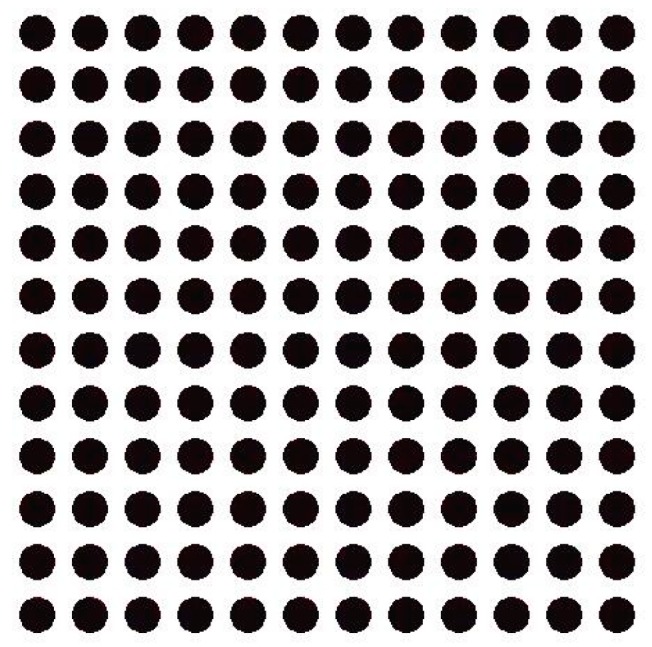
The results of segmentation.

Randomly selecting a reflected light image measuring 12 × 12, the results of segmentation are shown in Figure 13.

The experimental results demonstrate that the algorithm can eliminate bright spots on the right side of cells and segment the dots into the effective information region. The algorithm also improves the calculation accuracy of the gray value for a follow-up operation.

Finally, the total time of extracting the dots of a reflected light image has also been calculated. From the tilt RGB image to complete segmentation of the dots to be measured, the average time of dots extraction with nine pictures measuring 6 × 6 was 1.79 s, and the average time of dots extraction with nine pictures measuring 12 × 12 was 4.71 s. 

## 5. Conclusions

In this paper, research was conducted about dots extraction for PSi microarray images. First, a novel pretreatment method was presented according to the characteristics of the reflected light image and the relationship between the background and array cells. In the HSV space, the value component and saturation component are separately processed. The contour of cells can be segmented by the processing results of two components. Second, according to the geometric characteristics of the microarray image, a tilt correction algorithm was proposed based on MBR. The performance of this algorithm surpassed the traditional correction algorithms when applied to reflected light images. The principle of the algorithm is simple, and it has high calculation speed. Third, a segmentation algorithm is proposed for the requirement of image analysis in the reflected light image, which can eliminate the influence of gray value calculation caused by the edge and the bright spot of the array cell, and segment the required dots. In conclusion, the method proposed in this paper can avoid the influence of complex background and be utilized in the fast, accurate dots extraction of the PSi microarray reflected light image.

## Figures and Tables

**Table 1:** Data statistics of nine pictures by 6 × 6.

Algorithm	Average Error (°)	Average Time (s)
Radon transform	0.17	8.61
MBR correction	0.16	1.13

**Table 2:** Data statistics of nine pictures by 12 × 12.

Algorithm	Average Error (°)	Average Time (s)
Radon transform	0.10	34.38
MBR correction	0.06	1.70

**Table 3:** Segmentation time.

Sample	Average Size (pixel)	Average Time (s)
6 × 6	283 × 287	1.51
12 × 12	567 × 576	3.88
